# Relationship between clinical features and droplet digital PCR copy number in non-HIV patients with pneumocystis pneumonia

**DOI:** 10.1186/s12879-023-08580-7

**Published:** 2023-11-27

**Authors:** Wenjie Bian, Yu Xie, Ying Shang, Lili Zhao, Zhengwu Yang, Xinqian Ma, Yukun He, Wenyi Yu, Wen Xi, Donghong Yang, Fang Wang, Yanwen Chen, Pihua Gong, Zhancheng Gao

**Affiliations:** 1 Department of Respiratory and Critical Care Medicine, Peking University People’s Hospital, No.11, Xizhimen South Street, Beijing, China; 2 Department of Respiratory Medicine, Shanghai Ninth People’s Hospital, Shanghai Jiao Tong University School of Medicine, Shanghai, China

**Keywords:** Pneumocystis pneumonia, ddPCR copy number, BALF, mNGS

## Abstract

**Objective:**

Droplet digital PCR (ddPCR) is a novel assay to detect pneumocystis jjrovecii (*Pj*) which has been defined to be more sensitive than qPCR in recent studies. We aimed to explore whether clinical features of pneumocystis pneumonia (PCP) were associated with ddPCR copy numbers of *Pj*.

**Methods:**

A total of 48 PCP patients were retrospectively included. *Pj* detection was implemented by ddPCR assay within 4 h. Bronchoalveolar fluid (BALF) samples were collected from 48 patients with molecular diagnosis as PCP via metagenomic next generation sequencing (mNGS) or quantitative PCR detection. Univariate and multivariate logistic regression were performed to screen out possible indicators for the severity of PCP. The patients were divided into two groups according to ddPCR copy numbers, and their clinical features were further analyzed.

**Results:**

*Pj* loading was a pro rata increase with serum (1,3)-beta-D glucan, D-dimmer, neutrophil percentage, procalcitonin and BALF polymorphonuclear leucocyte percentage, while negative correlation with albumin, PaO2/FiO2, BALF cell count, and BALF lymphocyte percentage. D-dimmer and ddPCR copy number of *Pj* were independent indicators for moderate/severe PCP patients with PaO2/FiO2 lower than 300. We made a ROC analysis of ddPCR copy number of *Pj* for PaO2/FiO2 index and grouped the patients according to the cut-off value (2.75). The high copy numbers group was characterized by higher level of inflammatory markers. Compared to low copy number group, there was lower level of the total cell count while higher level of polymorphonuclear leucocyte percentage in BALF in the high copy numbers group. Different from patients with high copy numbers, those with high copy numbers had a tendency to develop more severe complications and required advanced respiratory support.

**Conclusion:**

The scenarios of patients infected with high ddPCR copy numbers of Pj showed more adverse clinical conditions. Pj loading could reflect the severity of PCP to some extent.

## Strengths and Limitations

This is the first study to investigate the relationship between ddPCR copy number and the clinical characteristics of patients with PCP.

Droplet digital PCR was a more sensitve and accurate method than qPCR for detection of pathogens.

This was a retrospective study and we did not include more samples for it.

## Background

*Pneumocystis jirovecii (Pj)* is a ubiquitous opportunistic fungus that can cause life-threatening pneumocystis pneumonia (PCP) in immunocompromised hosts [1]. Owing to the application of highly active immunosuppressive therapy, PCP has become increasingly common among non-HIV-infected patients with hematologic disorders or autoimmune diseases (HIV- human immunodeficiency virus) [2, 3]. Patients may present with fever, cough, and dyspnea when infected with *Pj* [4, 5]. Severe cases can lead to respiratory failure and even death [6]. Therefore, rapid and accurate detection is critical in patients with PCP. Pathological diagnosis is the gold standard for PCP, and microscopy is far from optimal because of the high probability of false negatives. In recent years, molecular detection methods have gradually been developed to detect *Pj*. Polymerase chain reaction (PCR) assays, which mainly target the mitochondrial large subunit rRNA gene and the multicopy major surface glycoprotein gene family, make the detection of *Pj* more sensitive than microscopic detection [7, 8]. However, no commercial PCR kits have been approved for clinical use in China, and their sensitivity is unsatisfactory [9]. Metagenomic next-generation sequencing (mNGS) is a high-throughput molecular technology that can achieve unbiased pathogen detection in a single assay. Due to its high sensitivity, it has become increasingly popular in recent years [8] and can be used to identify different pathogens involved in infectious diseases [10, 11]. However, its use has been limited by the high cost.

Droplet digital PCR (ddPCR), based on water-oil emulsion droplet technology, is a novel technique that can accurately quantify trace specimens [12, 13]. The number of droplets was counted after amplification, according to which an estimation of template concentration was made based on Poisson’s Law of Small Numbers [14]. Compared with conventional molecular methods, ddPCR has the advantages of higher sensitivity and specificity, absolute quantification without a standard curve, better reproducibility, and better resistance to PCR inhibitors [13]. Many studies using ddPCR assays have shown high sensitivity and precision [15–17]. Currently, several studies have focused on ddPCR detection of *Pj* and achieved excellent results [14, 18]. However, data on ddPCR copy numbers and clinical features of PCP patients are limited. We hypothesized that the clinical manifestations in patients with PCP are related to ddPCR copy numbers. In this study, we used ddPCR to accurately quantify copy numbers of *Pj* in BALF samples of PCP patients. We analyzed the ddPCR copy numbers and clinical features of PCP in order to determine the relationship between them.

## Materials and methods

### Study participants

**Fig. 1:**
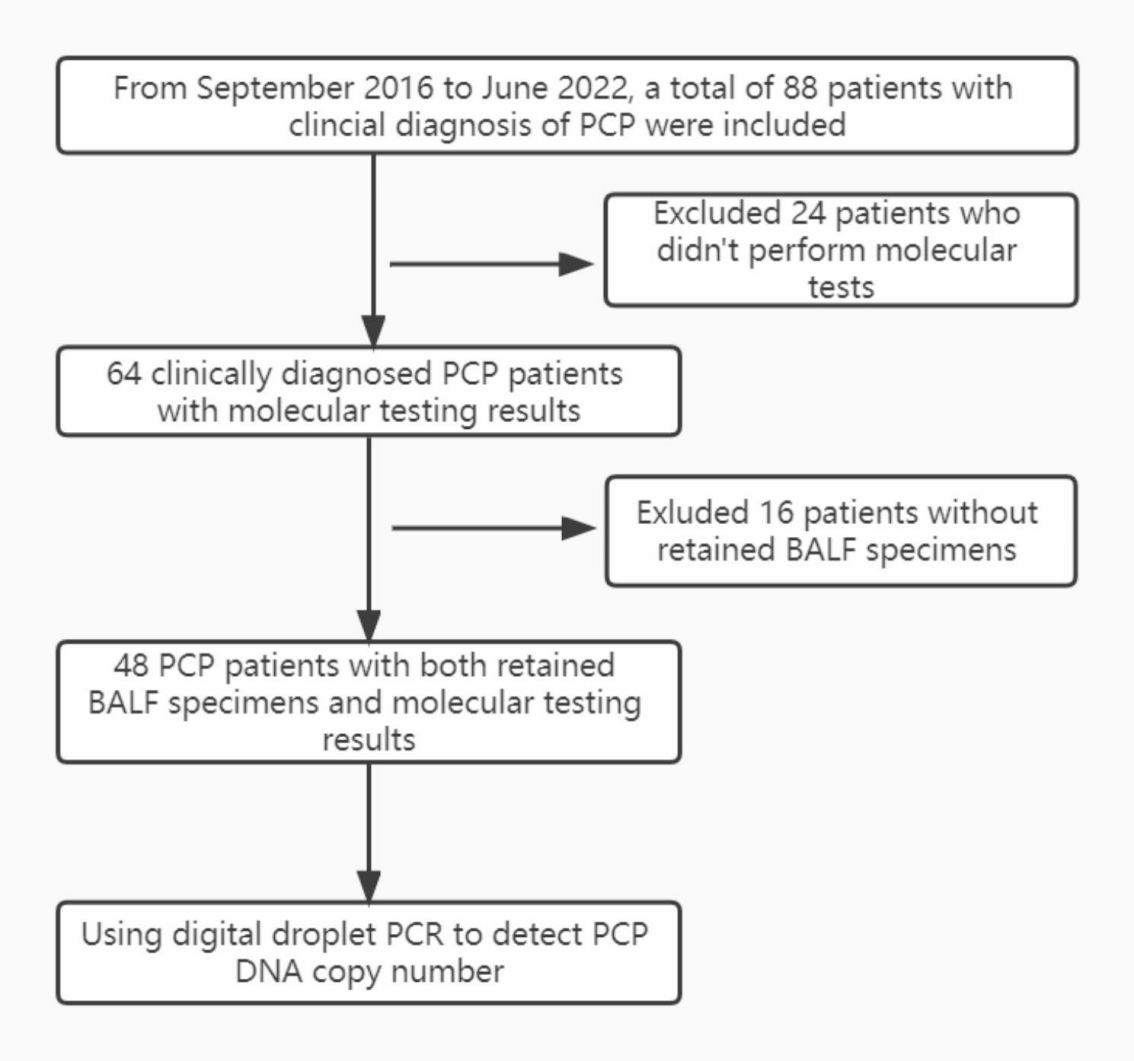
Study flowchart

This was a retrospective study performed on 48 non-HIV patients with clinical PCP diagnosis from September 2016 to June 2022 at Peking University People’s Hospital. Bronchoscopy was conducted within the initial five days of anti-PCP treatment commencement. The BALF specimens were all collected from patients during their hospitalization period, as part of clinical indications for bronchoscopy examinations, and were the remaining samples after necessary laboratory analysis for submission. The study was approved by the Institutional Review Board of Peking University People’s Hospital (No. 2022PHB457). The included patients needed to meet the following criteria: [1] Have at least one of the following severe immunosuppression conditions: primary immunodeficiencies, solid organ or bone marrow transplantation, hematologic neoplasia, or other diseases that cause deterioration in cellular immunity, including but not limited to radiotherapy and chemotherapy for cancer, autoimmune diseases of the connective tissue, asthma, and acute exacerbations chronic obstructive pulmonary disease (AECOPD), which required treatment with immunosuppressive medications such as prednisone at a dose of 0.3 mg/kg/day or its equivalent for a duration exceeding two weeks [2]. Patients who were diagnosed as PCP; [3] Patients with both retained BALF specimens and molecular testing results. Patients whose imaging and clinical manifestations do not correspond to PCP, which can be explained by other pathogens or colonization are excluded from the study. Forty patients who didn’t perform molecular detection (24 patient) or whose BALF specimen weren’t retained (16 patients) were excluded (Fig. 1).

### Definition

The clinical diagnosis of PCP requires meeting at least two of the following three criteria: ①respiratory symptoms including cough and/or dyspnoea, hypoxia, ②typical radiological picture such as ground glass opacity on a chest computer tomography scan, ③diffuse interstitial opacity on chest x-ray; receipt of a full course of treatment for PCP. A PCP case was considered if a patient meets the clinical diagnostic criteria and is either PCR or mNGS positive, or if they meet the clinical diagnostic criteria and show response to empirical treatment even with negative molecular testing. Colonization were described not fulfilling the clinical diagnostic criteria for PCP with positive PCR [8].

### Data collection

We recorded the following clinical and biological data for each patient: molecular detection results (mNGS or qPCR), underlying disease (hematological malignancies, autoimmune disease, renal diseases, cancer, and other diseases), vital signs, comorbidities, radiological signs (obtained by X-ray analysis or computed tomography scan), laboratory tests (routine blood tests, liver function, renal function, electrolytes, PCT, CRP, coagulation function, PaO2/FiO2, serum (1,3)-beta-D glucan (BDG), cytologic analysis for BALF), treatments and outcomes (Hospital stay, immunosuppressive medication, complications, oxygen support assays, and clinical outcome). Patients who had incomplete medical records, lost BALF samples or no molecular detection were excluded. BALF specimens were collected for qPCR or mNGS detection. Arterial blood gas results were collected on the same day of tracheoscopy. BDG results were included before or after BALF specimen were collected within 7 days. The requirement for written informed consent was waived off due to the retrospective nature of the study.

### DNA extraction

BALF specimens from patients were collected according to standard operating procedures. For DNA extraction, we used 1.5mL microcentrifuge tubes to contain a 0.6mL sample attached to a horizontal platform on a vortex mixer. The samples were agitated vigorously at 2,800–3,200 rpm for 15 min. Then they were centrifuged and the supernatant were discarded. Cells were resuspended in 100uL nucleic acid extraction reagent and transferred into another microcentrifuge tube with 1 g 0.5 mm glass beads. The samples were agitated vigorously at 2,800–3,200 rpm for 8 min and then had metal bath for 5 min at 95 °C. Nucleic Acid Extraction Kit (Capital Bio Technology) was used according to the manufacturer’s recommendation. DNA concentrations were determined with NanoDrop ONEc spectrophotometer (Thermo Fisher Scientific, USA).

### ddPCR detection of *Pj*

We used ddPCR via the Targeting One Digital PCR System (Targeting One, Beijing, China) for Pj detection. The primers and probes were designed according to previous research [14]. The sequences used were the mtLSU reference sequence of *Pj* and the human reference gene N-acetylglucosamine kinase (NAGK). The Forward primer of mtLSU reference sequence of *Pj* was GTATAGCACTGAATATCTCGAGGG. The Forward primer of mtLSU reference sequence of *Pj* was.

GAGCTTTAATTACTGTTCTGGGCT. The sequence of probe was FAM-TTCGACTATCTACCTTATCGC -MGB. The Forward primer of NAGK was AGATGCTGGGCAGACACATC. The Forward primer of NAGK was CCCACCTTCACTCCCACCT. The sequence of probe was HEX-AGCAGTGTTGCCCGAGATTG ACCC-BHQ1. The 30 µL PCR reaction mixture included 7.5 µL 4× SuperMix, 3 µL 10× primers and probes mix (the final concentrations of primers and probes were 600 and 300 nM, respectively), and up to 19.5 µL of the DNA template. We add 800ng genomic DNA sample as the template for PCR in the 30ul reaction mixture. The rest procedures were completed as described in the previous study. The reaction mixture of 30 µl was transferred into an 8-strip PCR tube and subjected to thermal cycling with the following conditions: initial denaturation at 95 °C for 10 min, followed by 45 amplification cycles involving denaturation at 94 °C for 30 s and annealing at 57 °C for 1 min. The reaction concluded with a final step at 12 °C. Following amplification, the amplified droplets within the 8-strip PCR tube were connected to a droplet detection chip and subsequently analyzed using the Chip Reader. When the copy number of the vector DNA found in that sample was greater than 5, the sample was considered positive and the measured copy number was reported.

### BALF qPCR and NGS

After DNA extraction from BALF samples, PCR detection was performed. The specific detection process and synthesis of primers were based on previous literature [19]. The NGS detection process was also conducted following the procedures outlined in previous literature [20].

### Statistical analysis

The severity of PCP was assessed based on the PaO2/FiO2 index when BALF specimen were collected and was categorized as mild (≥ 300 mmHg) or severe (< 300 mmHg) pneumonia. A log_10_-transformation (log_10_ [ddPCR copy number] and log_10_ [sequence number]) was applied to normalize the data. We made the univariate logistic regression analysis and found ddPCR copy number to be a risk factor for the severity of PCP according to PaO2/FiO2 index. Then we made a receiver operating characteristic (ROC) analysis and calculated the cutoff value. Finally, the patients were grouped into low copy numbers (n = 24) and high copy numbers (n = 24) groups according to the cutoff value. Finally, Continuous variables in a normal distribution were presented as the mean (± S.D.) while the median (25th–75th percentile) in a non-normal distribution. Continuous data with a non-normal distribution were analyzed by Wilcoxon rank-sum test while T test with a normal distribution. Categorical variables were analyzed by chi-squared test. A *p*-value < 0.05 was considered statistically significant. The correlations of different markers and ddPCR copy numbers were determined using the Spearman’s correlation for nonparametric data. As for sample size, we set 1-α to be 0.8, AUC to be 0.75, and Confidence Interval Width to be 0.2. Using he PASS 15 software, we determined that a sample size of 40 is necessary.

## Results

### Baseline characteristics of PCP patients

A total of 48 patients were enrolled in this study. As shown in Table 1, the median age of the subjects was 53 years, and 52% were men. The primary diseases were autoimmune and hematological diseases, accounting for 90% of all cases. Approximately 40% of the patients had comorbidities such as diabetes, hypertension, hyperlipidemia, and cardiovascular disease. Most patients had cough, fever, and dyspnea. Patients with moderate-to-severe PCP had fewer cardiovascular diseases and higher heart rates and higher ddPCR copy numbers. There were no significant differences between the mild and moderate/severe PCP patients regarding sex, age, primary diseases, fever, highest temperature, cough, and dyspnea.

**Table 1:** Baseline characteristics of PCP patients

	All patients(n = 48)	mild PCP patients (n = 24)	moderate/severe PCP patients (n = 24)	*p-*value
**characteristics**				
Age, years	52.52(± 16.417)	49.53(± 17.014)	54.48(± 16.008)	0.311
Sex				0.263
Men	25(52%)	8(12%)	17(71%)	
Women	23(48%)	11(46%)	12(50%)	
Primary Disease				
Autoimmune disease	24(50%)	10(42%)	14(58%)	0.768
Hemological disease	19(40%)	9(37%)	10(42%)	0.372
Others	5(10%)	0(0)	5(21%)	0.142
Any Comorbidity	19(40%)	8(12%)	11(46%)	0.772
Diabetes	11(23%)	4(17%)	7(29%)	1
Hypertension	10(21%)	3(12%)	7(29%)	0.739
Cardiovascular disease	5(10%)	5(21%)	0(0)	0.007
Hyperlipidemia	8(17%)	4(17%)	4(17%)	0.695
Signs and symptoms				
Fever	21(44%)	6(25%)	15(62%)	0.169
Highest temperature,℃				0.445
<37.3	27(56%)	13(54%)	14(58%)	
37.3–38.0	9(19%)	3(12%)	6(25%)	
38.1–39.0	10(21%)	2(8%)	8(33%)	
> 39.0	2(4%)	1(4%)	1(4%)	
Heart rate, /min	90(78–104)	82(78–92)	93(80–113)	0.02
Cough	32(67%)	15(62%)	17(71%)	0.251
Dyspnoea	24(50%)	9(37%)	15(62%)	0.768
ddPCR copy number	711.67(26.85-8550.17)	43.95(12.35–584.50)	2439.75(263.37-11785.30)	0.001

Data were presented as the mean (± S.D.) or median (25th–75th percentile). P-values by Wilcoxon rank-sum test or T test. Categorical variables were analyzed by chi-squared test.

### Assessment of the ddPCR-based detection of PCP

**Fig. 2:**
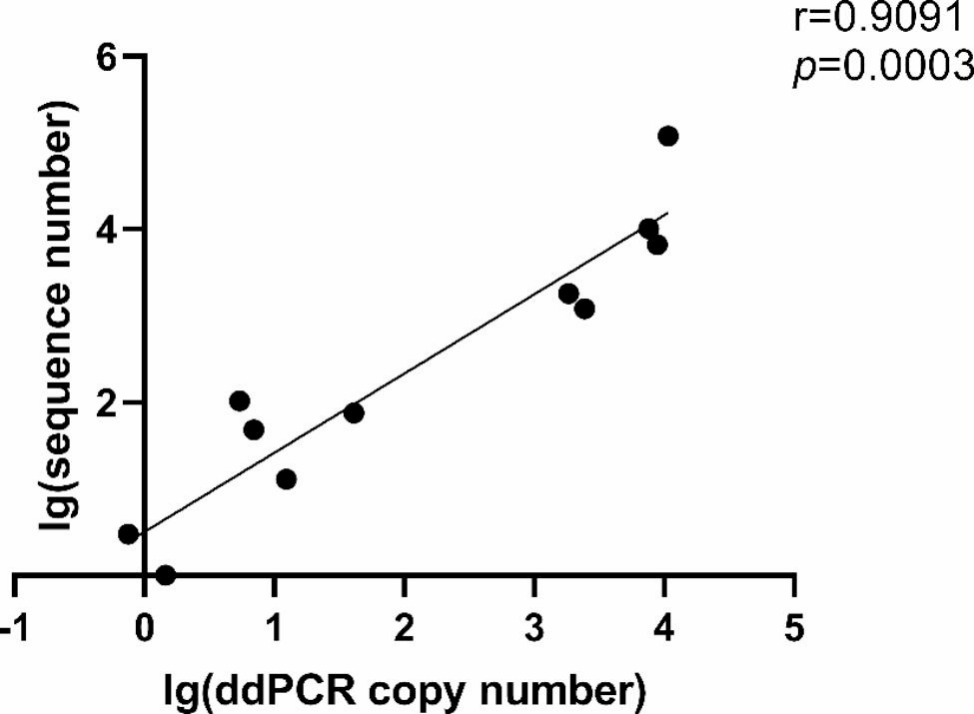
Correlation analysis between the mNGS sequence number and the *Pj* load of ddPCR in BALF. The mNGS sequence number had a good correlation with the *Pj* load of ddPCR in BALF.

First, we performed the ddPCR assay. Twenty-three specimens were detected using mNGS, while 25 were detected using PCR. Among the 23 patients who underwent mNGS assays, two patients had negative ddPCR results, which were positive on mNGS, and one had a negative mNGS and positive ddPCR. Of the 25 patients who underwent PCR assays, two patients had negative results from the PCR assay but positive ddPCR results and one had a positive result from the PCR assay and a negative ddPCR. The detailed data are presented in Table 2. Eleven patients had data on mNGS sequence numbers, and we performed a correlation analysis with ddPCR copy numbers. As shown in Fig. 2, there was a significant positive correlation between the two.

**Table 2:** The discrepancies among different assays

Patients	mNGS	PCR	ddPCR	Copy number/sequence number
Patient 1	positive	/	negative	1.45/1
Patient 2	positive	/	negative	0.75/3
Patient 3	negative	/	positive	17.05/null
Patient 4	/	positive	negative	1.55
Patient 5	/	ngative	positive	181.45
Patient 6	/	negative	positive	287.75

mNGS metagenomics next generation sequencing, ddPCR droplet digital polymerase chain reaction.

### Correlations between the ddPCR copy number and clinical indicators

**Fig. 3:**
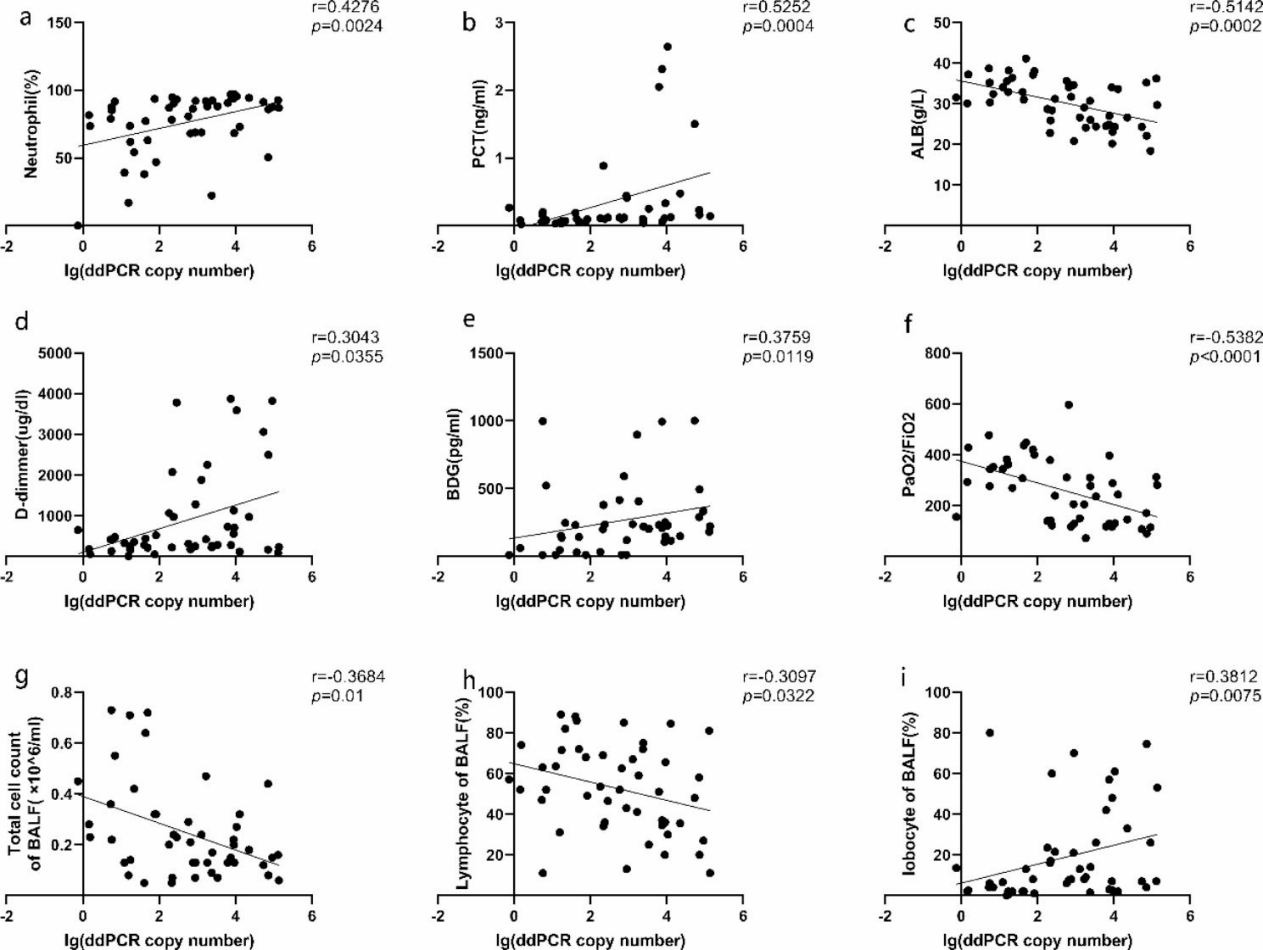
Correlation of different clinical indicators with the ddPCR copy number in 48 patients of PCP. We found that the ddPCR copy number had significant correlation with neutrophil percentage (**a**), PCT (**b**), ALB (**c**), d-dimmer (**d**), BDG (**e**), PaO2/FiO2 (**f**), BALF total cell count (**g**), BALF lymphocyte percentage (**h**), and BALF locbocyte percentage (**i**). MPN, polymorphonuclear leucocyte

We performed a correlation analysis between copy numbers and different indicators (Fig. 3). The copy number was positively correlated with serum [1, 3]-β-D glucan, d-dimer, neutrophil percentage, procalcitonin (PCT), and BALF polymorphonuclear leukocyte percentage while negatively correlated with albumin, PaO_2_/FiO_2_, BALF cell count, and BALF lymphocyte percentage.

### Statistical analysis of risk factors for PCP severity and ROC analysis of ddPCR copy numbers

**Fig. 4:**
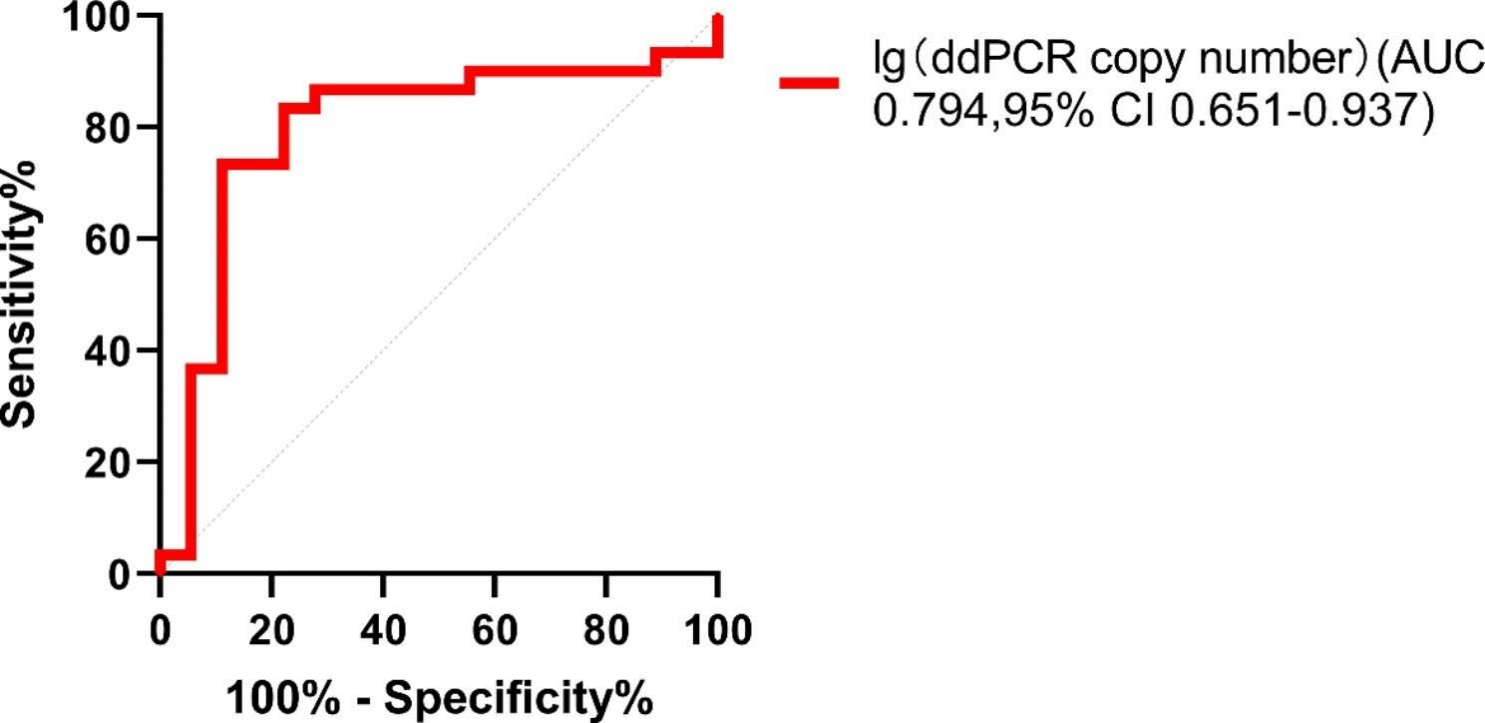
ROC curve analysis of ddPCR copy number for severity of PCP. AUC was 0.794 and cut-off value of lg (copy number) was 2.85

Independent indicators were introduced into univariate logistic regression analysis (Table 3). The results showed that high levels of neutrophil percentage, d-dimer, and lg (copy number) were associated with significantly high-risk ratios of low PaO_2_/FiO_2_, while high levels of serum albumin (ALB) were associated with significantly low-risk ratios. When the significant independent variables were integrated into a multivariate logistic regression analysis, only D-dimer [hazard ratio (95% confidence interval (CI)):1.004 (1.000-1.009)] and lg (copy number) [hazard ratio (95% CI):1.787 (1.036–3.082)] were found to be significant independent predictors of PCP severity (*p* = 0.034 and *p* = 0.037, respectively). To investigate whether the ddPCR copy numbers were related to the clinical features of PCP, we performed a ROC analysis between lg (copy number) and PaO_2_/FiO_2_ index (Fig. 4). The AUC was 0.794. Finally, we determined the cut-off value of lg (copy number) to be 2.85.

**Table 3:** Univariate and multivariate analysis of risk factors for PCP severity

	Univariate analysis		Multivariate analysis	
	Hazard ratio (95% CI)	*p* value	Hazard ratio (95% CI)	*p* value
Age	0.981(0.947–1.017)	0.305		
White blood cell count	0.974(0.856–1.174)	0.974		
Neutrophil percent	0.969(0.940–0.999)	**0.040**		
Hemoglobin level	1.018(0.992–1.046)	0.173		
Platelet count	1.006(0.999–1.012)	0.079		
D-dimmer	0.996(0.993–0.999)	**0.017**	0.996(0.991-1.000)	**0.034**
Albumin	1.139(1.118–1.556)	**0.001**		
Blood urea nitrogen	0.985(0.935–1.037)	0.561		
BDG	0.999(0.997–1.002)	0.645		
lg(copy number)	0.482(0.292–0.797)	**0.004**	0.560(0.325–0.965)	**0.037**

BDG (1,3)-beta-D glucan

### Comparison of clinical manifestions in high and low ddPCR copy number groups

The 48 patients enrolled from September 2016 to June 2022 were divided into low and high copy number groups according to the cut-off value of the ROC analysis. Table 4 shows the baseline characteristics and laboratory results of the patients with PCP with low or high ddPCR copy numbers. More patients with other diseases, such as tumors or glomerular diseases, were in the high-copy number group. No statistical difference was observed between the two groups in terms of age, sex, comorbidities, temperature, heart rate, cough, or dyspnea. The high copy number group had higher levels of inflammatory markers, such as neutrophil percentage and PCT. Other indicators in blood showed that the high copy number group exhibited lower levels of platelet count and ALB but higher levels of BDG. The PaO_2_/FiO_2_ index was calculated and found to be significantly lower in the high copy number group. Compared to the low copy number group, there were lower total cell counts and higher levels of polymorphonuclear leukocytes (PMN) in BALF in the high copy number group. Next, we compared the treatment outcomes between the two groups. Compared to patients with high copy numbers, those with low copy numbers tend to develop more severe complications and require advanced respiratory support. Although the difference was not statistically significant, the median length of hospitalization was longer in the high copy number group. We did not find any difference in immunosuppressive therapy methods between the two groups.

**Table 4:** Clinical manifestions of PCP patients with low and high copies in hospital

Parameters	PCP patients with low copies	n	PCP patients with high copies	n	*p*-value
**Characteristics**					
Age, years	54.29(± 16.578)	24	50.75(± 16.414)	24	0.796
Sex		24		24	
Men	12(50%)	24	13(54%)	24	0.773
Women	12(50%)	24	11(46%)	24	
Primary Disease					
Autoimmune disease	12(50%)	24	12(50%)	24	1
Hematological disease	12(50%)	24	7(29%)	242	0.14
Others	0(0)	24	5(21%)	4	0.05
Any Comorbidity	8(33%)	24	11(46%)	24	0.376
Diabetes	5(21%)	24	6(25%)	24	0.731
Hypertension	3(12%)	24	7(29%)	24	0.286
Cardiovascular disease	3(12%)	24	2(8%)	24	1
Hyperlipidemia	3(12%)	24	5(21%)	24	0.699
**Signs and symptoms**					
Fever	10(42%)	24	11(46%)	24	0.771
Highest temperature,℃					
< 37.3	14(58%)	24	13(54%)	24	0.386
37.3–38.0	3(12%)	24	6(25%)	24	
38.1–39.0	5(21%)	24	5(21%)	24	
> 39.0	2(8%)	24	0(0)	24	
Heart rate, /min	88(78–104)	24	92(82–104)	24	0.430
Cough	18(75%)	24	14(58%)	24	0.221
Dyspnoea	10(42%)	24	14(58%)	24	0.248
**Blood routine**					
White blood cell count, ×10^9^/L	7.0(5.32–9.96)	24	7.1(5.69–9.52)	24	0.813
Neutrophil percent, %	77.6(56.1–87.3)	24	89.3(76.2–93.4)	24	**0.008**
Hb, g/L	106(± 24.853)	24	106(± 22.962)	24	0.934
PLT count, ×10^9^/L	198(120–247)	24	128(87–193)	24	**0.047**
> 100	4(17%)		7(29%)		0.492
< 100	20(83%)		17(71%)		
**Coagulant function**					
Prothrombin time, s	11.7(11.1–13.7)	24	12.1(11.3–13.4)	24	0.563
D-dimer, ng/ml	318(169–506)	24	628(232–2157)	24	0.061
**Biochemical values**					
ALT, U/L	21(14–44)	24	29(15–52)	24	0.522
AST, U/L	32(18–36)	24	29(22–56)	24	0.556
ALB, g/L	33(30–36)	24	26(24–31)	24	**< 0.0001**
Blood glucose, mmol/L	5.25(4.78–5.91)	24	5.87(4.83–8.48)	23	0.183
BUN, mmol/L	5.58(3.86–5.16)	24	6.52(4.82–8.71)	24	**0.003**
Scr, µmol/L	74(58–91)	24	60(46–108)	24	0.529
**Arterial blood gas**					
PH	7.45(7.43–7.48)	24	7.47(7.44–7.49)	24	0.369
PO2, mmHg	86(72–113)	24	81(64–99)	24	0.212
PCO2, mmHg	33(± 11.683)	24	34(± 21.925)	24	0.569
HCO3-, mmol/L	24.4(21.3–25.7)	24	26.5(20.9–28.4)	24	0.140
PaO2/FiO2	348(271–415)	24	147(117–268)	24	**< 0.0001**
**Cell classification and count of alveolar lavage fluid**					
Total cell count, ×10^6^/ml	0.26(0.15–0.44)	24	0.14(0.12–0.21)	24	**0.012**
Macrophage, %	32(± 29.2)	24	28(± 19.1)	24	0.934
Lymphocyte, %	59(± 19.3)	24	47(± 22.9)	24	0.056
polymorphonuclear leucocyte, %	6(2.0-15.4)	24	13(7–46)	24	**0.018**
**Other laboratory blood tests**					
CRP, mg/dl	27.8(15–77)	23	33.6(19–125)	23	0.517
PCT, mg/dl	0.08(0.06–0.14)	21	0.33(0.13–2.05)	19	**< 0.0001**
BDG, pg/ml	136.9(10.0-240.5)	21	229.1(156.9-471.6)	24	**0.013**
**Treatments and outcomes**					
Hospital stays	16(13–32)	24	26(15–37)	24	0.117
Complications	2(8%)	24	10(42%)	24	**0.02**
Acute respiratory distress syndrome	1(4%)	24	4(17%)	24	0.348
Acute cardiac injury	0(0)	24	2(8%)	24	0.489
Acute kidney injury	1(4%)	24	5(21%)	24	0.188
Shock	1(4%)	24	3(12%)	24	0.609
Oxygen support		24		24	
None	11(46%)	24	3(12%)	24	**0.027**
Nasal cannula	10(42%)	24	13(54%)	24	
Mechanical ventilation	3(12%)	24	8(33%)	24	
Immunosuppressive therapy		24		24	
None	1(4%)	24	0(0)	24	0.719
Glucocorticoid	5(21%)	24	4(17%)	24	
Glucocorticoid combined with immunosuppressive agent	16(67%)	24	17(71%)	24	
Chemotherapeutic agents	2(8%)	24	3(12%)	24	
Prognosis		24		24	1
Discharge	23(96%)	24	23(96%)	24	
Death	1(4%)	24	1(4%)	24	

Continuous data were presented as the mean (± S.D.) or median (25th–75th percentile). P-values by Wilcoxon rank-sum test or T test. Categorical variables were analyzed by chi-squared test. ALT alaninetransaminase, AST aspartate transaminase, ALB albumin, PMN polymorphonuclear leucocyte, CRP C-reactive protein, PCT procalcitonin, BDG [1, 3]-beta-D glucan

## Discussion

To our knowledge, this is the first study to investigate the relationship between ddPCR copy number and the clinical characteristics of patients with PCP. In this study, we found that ddPCR copy number was closely related to the clinical features of PCP and could reflect the severity of PCP to an extent.

As a novel assay, ddPCR has revealed good diagnostic performance for PCP. A study compared ddPCR with quantitative PCR and found the same results for 95.12% of BALF samples, while the remaining samples tested positive using ddPCR but negative using qPCR. mNGS results corroborated these inconsistent results. The authors also found that the cycle threshold value of qPCR was highly correlated with the ddPCR copy number (R^2^ = 0.84) [14]. In another study, using a cut-off value of > 4.5 copies/uL, the ddPCR assay exhibited a sensitivity of 100% and specificity of 94.3% in non-HIV patients with PCP [18]. In our study, mNGS yielded highly consistent results for BALF specimens (R^2^ = 0.81). This indicated the diagnostic accuracy of the ddPCR system. Three samples tested negative by ddPCR, and the mNGS sequence numbers of the two were 1 and 3, respectively. This implied that the samples had a low fungal load. Considering the small sample size of BALF and the long storage time of these specimens, the expected effect of ddPCR could not be detected. Despite this, the novel assay exhibited a promising performance.

Since ddPCR enables precise quantification of nucleic acids, we performed Spearman correlation analysis between copy numbers and different clinical indicators. Neutrophil percentage, PCT, and d-dimer levels were positively correlated, while albumin was negatively correlated with ddPCR copy number. Neutrophils are important factors in pulmonary inflammation and can lead to lung damage when excessively activated [21]. PCT is a useful marker for discriminating between viral and bacterial pneumonia [22]. D-dimer can indicate excessive inflammation and vascular endothelial injury [23]. Hypoalbuminemia can reflect the severity of inflammation and is an independent risk factor for mortality [24]. Therefore, the high fungal burden in BALF was associated with severe inflammation and poor prognosis in patients with PCP to some extent. The oxygenation index was negatively correlated with the ddPCR copy number in our results. A low oxygenation index indicated a more severe clinical condition. This means that copy number was correlated with the severity of PCP. BDG is an important component of the cell wall of most fungi, including *Pj*, making it a significant diagnostic indicator of PCP [25]. Some studies have investigated whether serum BDG could be used to estimate the fungal burden, but the results were inconsistent in people with different diseases [26, 27]. A study used qPCR assays to detect *Pj* in BALF, performed correlation analysis for Ct value and serum BDG, and found a weak correlation between them [26]. From our findings, ddPCR copy number significantly correlated with BDG. This was because the ddPCR assay accurately detected the fungal load. The BALF total cell count and BALF lymphocyte percentage were negatively correlated with the ddPCR copy number, while the BALF PMN percentage was positively correlated with it. PCP patients are prone to a decrease in the total cell count and lymphocytes due to the use of immunosuppressive agents. Reduced CD8 + T cell levels can be used to predict the adverse development of PCP in immunosuppressed hosts [28]. The BALF PMN percentage increased due to the decreased lymphocyte percentage.

PCP is a life-threatening condition in immunosuppressed hosts. Many studies have investigated indicators for predicting the prognosis of PCP. One study found that platelet count and γ-globulin could be risk factors in the HIV group in the multivariate analysis, while albumin could be a risk factor in the non-HIV group in the univariate analysis [29]. In another study, mixed infection and elevation of NLR were considered independent risk factors for poor prognosis of non-HIV PCP patients diagnosed by mNGS [10]. We performed univariate and multivariate regression analyses for PaO_2_/FiO_2_ index and found ddPCR copy number and D-dimer to be risk factors. In contrast to previous studies with outcome measures of mortality, we defined an oxygenation index before tracheoscopy detection with less than 300 as the outcome. The two outcome indicators were consistent. For example, plasma interleukin (IL)-6 and IL-8 levels were significantly higher in PCP patients with lower oxygenation as well as in non-survivors [30]. Studies have evaluated the relationship between Ct-value and PCP severity but found no significant difference between the severe and mild PCP groups. Nonetheless, a trend of higher Ct values in the mild group was found [8]. The accurate quantification of ddPCR enabled us to further investigate their relationship. Individuals with a low PaO_2_/FiO_2_ index had significantly higher ddPCR copy numbers. Further analysis revealed that a high fungal load in the BALF can be an independent risk factor for disease severity. To deeply explore the relationship between ddPCR copy number and clinical features, we performed ROC analysis for the copy number and divided the patients into two groups according to the cut-off value. We normalized the data by applying a log_10_-transformation (log_10_-[ddPCR copy number]). Differences in laboratory results and treatments were observed between the groups. High copy numbers were associated with more severe clinical presentations, such as higher inflammatory markers, more advanced oxygen support, and more severe complications. Based on the above findings, we concluded that the ddPCR copy number could reflect the severity of PCP. The treatment outcomes also showed differences between the two groups. The high copy number group had a longer hospital stay and more severe complications than the low copy number group. Univariate analysis showed that mechanical ventilation could be a risk factor for patient mortality [31]. These results indicate that the clinical condition was worse in the high copy number group. Therefore, the ddPCR copy number may help to evaluate clinical severity in patients with PCP. Categorizing patients according to copy numbers offers valuable insights for enhancing the management of PCP cases. Those with elevated copy numbers necessitate swift and comprehensive anti-PCP treatment, coupled with vigilant monitoring of abnormal laboratory parameters. Furthermore, their susceptibility to advancing into respiratory failure underscores the critical importance of close surveillance. We can include more patients to further confirm this finding and investigate whether the ddPCR copy number of *Pj* in BALF can reflect disease mortality in future studies.

There were several limitations to our study, along with opportunities for further studies. Firstly, the sample size was not sufficiently large. Secondly, not all patients are eligible for bronchoscopy. This study has exclusively explored BALF specimens, but future investigations can encompass additional respiratory specimens such as sputum. This would allow for broader applicability of our findings. Thirdly, it’s worth noting that in this study, the majority of patients initiated empirical treatment before undergoing bronchoscopy. This factor could potentially influence our quantitative results.

## Conclusions

In conclusion, this study addressed the clinical features and ddPCR copy numbers in patients with PCP. Our data suggest that ddPCR copy number has a good correlation with many laboratory indicators such as albumin, PCT, d-dimer, BDG, and BALF lymphocytes and can, to an extent, reflect PCP severity.

## Data Availability

The original contributions presented in the study are included in the article, further inquiries can be directed to the corresponding authors.

## Abbreviations

BALF: Bronchoalveolar fluid
ddPCR: Droplet digital PCR
*Pj*: Pneumocystis jjrovecii
mNGS: Metagenomic next generation sequencing
PCP: Pneumocystis pneumonia
PCR: Polymerase chain reaction
PCT: Procalcitonin
ALB: Albumin
CI: Confidence interval
BDG: (1,3)-beta-D glucan
PMN: Polymorphonuclear leukocytes
CRP: C-reactive protein
ALT: Alaninetransaminase
AST: Aspartate transaminase

## References

[1] Fortea JI, Cuadrado A, Puente Á, Álvarez Fernández P, Huelin P, Álvarez Tato C, et al. Is routine Prophylaxis Against Pneumocystis jirovecii needed in liver transplantation? A retrospective single-centre experience and current prophylaxis strategies in Spain. J Clin Med. 2020;9(11). 10.3390/jcm9113573. 10.3390/jcm9113573 PMC7694638 33171962

[2] Liu CJ, Lee TF, Ruan SY, Yu CJ, Chien JY, Hsueh PR (2019). Clinical characteristics, treatment outcomes, and prognostic factors of Pneumocystis pneumonia in non-HIV-infected patients. Infect Drug Resist.

[3] Lee WS, Hsueh PR, Hsieh TC, Chen FL, Ou TY, Jean SS (2017). Caspofungin salvage therapy in Pneumocystis jirovecii pneumonia. J Microbiol Immunol Infect.

[4] Brakemeier S, Pfau A, Zukunft B, Budde K, Nickel P (2018). Prophylaxis and treatment of Pneumocystis Jirovecii pneumonia after solid organ transplantation. Pharmacol Res.

[5] Kim TO, Lee JK, Kwon YS, Kim YI, Lim SC, Kim MS (2021). Clinical characteristics and prognosis of patients with pneumocystis jirovecii pneumonia without a compromised illness. PLoS ONE.

[6] Enomoto T, Azuma A, Kohno A, Kaneko K, Saito H, Kametaka M (2010). Differences in the clinical characteristics of Pneumocystis jirovecii pneumonia in immunocompromized patients with and without HIV infection. Respirology.

[7] Salsé M, Mercier V, Carles MJ, Lechiche C, Sasso M (2021). Performance of the RealStar(®) pneumocystis jirovecii PCR kit for the diagnosis of Pneumocystis pneumonia. Mycoses.

[8] Hammarström H, Grankvist A, Broman I, Kondori N, Wennerås C, Gisslen M (2019). Serum-based diagnosis of Pneumocystis pneumonia by detection of Pneumocystis jirovecii DNA and 1,3-β-D-glucan in HIV-infected patients: a retrospective case control study. BMC Infect Dis.

[9] Del Corpo O, Butler-Laporte G, Sheppard DC, Cheng MP, McDonald EG, Lee TC (2020). Diagnostic accuracy of serum (1–3)-β-D-glucan for Pneumocystis jirovecii pneumonia: a systematic review and meta-analysis. Clin Microbiol Infect.

[10] Duan J, Gao J, Liu Q, Sun M, Liu Y, Tan Y (2022). Characteristics and prognostic factors of Non-HIV immunocompromised patients with Pneumocystis Pneumonia diagnosed by Metagenomics Next-Generation sequencing. Front Med (Lausanne).

[11] Goldberg B, Sichtig H, Geyer C, Ledeboer N, Weinstock GM (2015). Making the Leap from Research Laboratory to Clinic: Challenges and Opportunities for Next-Generation sequencing in Infectious Disease Diagnostics. mBio.

[12] Gaňová M, Zhang H, Zhu H, Korabečná M, Neužil P (2021). Multiplexed digital polymerase chain reaction as a powerful diagnostic tool. Biosens Bioelectron.

[13] Kojabad AA, Farzanehpour M, Galeh HEG, Dorostkar R, Jafarpour A, Bolandian M (2021). Droplet digital PCR of viral DNA/RNA, current progress, challenges, and future perspectives. J Med Virol.

[14] Yi J, Wang N, Wu J, Tang Y, Zhang J, Zhu L (2021). Development of a Droplet Digital polymerase chain reaction for sensitive detection of Pneumocystis jirovecii in respiratory tract specimens. Front Med (Lausanne).

[15] Li H, Bai R, Zhao Z, Tao L, Ma M, Ji Z, et al. Application of droplet digital PCR to detect the pathogens of infectious diseases. Biosci Rep. 2018;38(6). 10.1042/bsr20181170. 10.1042/BSR20181170 PMC6240714 30341241

[16] Chen B, Xie Y, Zhang N, Li W, Liu C, Li D (2021). Evaluation of Droplet Digital PCR assay for the diagnosis of Candidemia in blood samples. Front Microbiol.

[17] Zheng Y, Jin J, Shao Z, Liu J, Zhang R, Sun R (2021). Development and clinical validation of a droplet digital PCR assay for detecting Acinetobacter baumannii and Klebsiella pneumoniae in patients with suspected bloodstream infections. Microbiologyopen.

[18] Jitmuang A, Nititammaluk A, Boonsong T, Sarasombath PT, Sompradeekul S, Chayakulkeeree M (2021). A novel droplet digital polymerase chain reaction for diagnosis of Pneumocystis pneumonia (PCP)-a clinical performance study and survey of sulfamethoxazole-trimethoprim resistant mutations. J Infect.

[19] Wu Y, Wang F, Wang C, Tang X, Liu X, Li S (2021). Detection of Pneumocystis jirovecii and Toxoplasma gondii in patients with lung infections by a duplex qPCR assay. PLoS Negl Trop Dis.

[20] Liu L, Yuan M, Shi Y, Su X (2021). Clinical performance of BAL Metagenomic Next-Generation sequence and serum (1,3)-β-D-Glucan for Differential diagnosis of Pneumocystis jirovecii Pneumonia and Pneumocystis jirovecii Colonisation. Front Cell Infect Microbiol.

[21] Bordon J, Aliberti S, Fernandez-Botran R, Uriarte SM, Rane MJ, Duvvuri P (2013). Understanding the roles of cytokines and neutrophil activity and neutrophil apoptosis in the protective versus deleterious inflammatory response in pneumonia. Int J Infect Dis.

[22] Giulia B, Luisa A, Concetta S, Bruna LS, Chiara B, Marcello C (2015). Procalcitonin and community-acquired pneumonia (CAP) in children. Clin Chim Acta.

[23] Huang X, Li D, Liu F, Zhao D, Zhu Y, Tang H (2021). Clinical significance of D-dimer levels in refractory Mycoplasma pneumoniae pneumonia. BMC Infect Dis.

[24] Zou XL, Feng DY, Wu WB, Yang HL, Zhang TT (2021). Blood urea nitrogen to serum albumin ratio independently predicts 30-day mortality and severity in patients with Escherichia coli bacteraemia. Med Clin (Barc).

[25] Watanabe T, Yasuoka A, Tanuma J, Yazaki H, Honda H, Tsukada K (2009). Serum (1–>3) beta-D-glucan as a noninvasive adjunct marker for the diagnosis of Pneumocystis pneumonia in patients with AIDS. Clin Infect Dis.

[26] Mercier T, Aissaoui N, Gits-Muselli M, Hamane S, Prattes J, Kessler HH, et al. Variable correlation between Bronchoalveolar Lavage Fluid Fungal load and Serum-(1,3)-β-d-Glucan in patients with Pneumocystosis-A Multicenter ECMM Excellence Center Study. J Fungi (Basel). 2020;6(4). 10.3390/jof6040327. 10.3390/jof6040327 PMC7711754 33271743

[27] Szvalb AD, Malek AE, Jiang Y, Bhatti MM, Wurster S, Kontoyiannis DP (2020). Serum (1,3)-Beta-d-Glucan has suboptimal performance for the diagnosis of Pneumocystis jirovecii pneumonia in cancer patients and correlates poorly with respiratory burden as measured by quantitative PCR. J Infect.

[28] Tang G, Tong S, Yuan X, Lin Q, Luo Y, Song H (2021). Using routine laboratory markers and immunological indicators for Predicting Pneumocystis jiroveci Pneumonia in Immunocompromised Patients. Front Immunol.

[29] Ewig S, Bauer T, Schneider C, Pickenhain A, Pizzulli L, Loos U (1995). Clinical characteristics and outcome of Pneumocystis carinii pneumonia in HIV-infected and otherwise immunosuppressed patients. Eur Respir J.

[30] Sun J, Su J, Xie Y, Yin MT, Huang Y, Xu L (2016). Plasma IL-6/IL-10 ratio and IL-8, LDH, and HBDH Level predict the severity and the risk of death in AIDS patients with Pneumocystis Pneumonia. J Immunol Res.

[31] Torres HA, Chemaly RF, Storey R, Aguilera EA, Nogueras GM, Safdar A (2006). Influence of type of cancer and hematopoietic stem cell transplantation on clinical presentation of Pneumocystis jiroveci pneumonia in cancer patients. Eur J Clin Microbiol Infect Dis.

